# The Application of Wearable Technology in Surgery: Ensuring the Positive Impact of the Wearable Revolution on Surgical Patients

**DOI:** 10.3389/fsurg.2014.00039

**Published:** 2014-09-19

**Authors:** Jesse Alan Slade Shantz, Christian J. H. Veillette

**Affiliations:** ^1^University of California, San Francisco, CA, USA; ^2^University of Toronto Orthopaedic Sports Medicine, Toronto, ON, Canada; ^3^University Health Network, Toronto, ON, Canada

**Keywords:** wearable technology, wearable devices, augmented reality, outcome assessment (health care), surgery, computer-assisted

## Abstract

Wearable technology has become an important trend in consumer electronics in the past year. The miniaturization and mass production of myriad sensors have made possible the integration of sensors and output devices in wearable platforms. Despite the consumer focus of the wearable revolution some surgical applications are being developed. These fall into augmentative, assistive, and assessment functions and primarily layer onto current surgical workflows. Some challenges to the adoption of wearable technologies are discussed and a conceptual framework for understanding the potential of wearable technology to revolutionize surgical practice are presented.

## Introduction

An emerging industry, wearable technology, has made possible the integration of technology into many facets of daily life, including surgical practice. The trend has been supported by the miniaturization of the components necessary for the collection, storage, processing, sharing, and presentation of data. In particular, the ubiquity of accelerometers (1) and gyroscopes has spurred the development of myriad activity trackers (2, 3). As a result of the unique requirements of surgeons working in a sterile environment wearable technology use cases have appeared in the media (4, 5). The following review will provide insight on the roles wearable technology could play in surgical practice and discuss the barriers and key success factors for adoption of these potentially disruptive technologies.

### Defining wearable technology

Wearable technology or wearables are small electronic devices embedded into items that attach to the body and possess computational capability (6). Devices can be integrated into clothing (7), recognizable personal accessories (e.g., glasses, contact lenses, watches) or additional devices (e.g., pocket device to count steps).

Wearable technologies typically have several distinct, but connected, components. The hardware itself consists of a combination of sensors, a display, a processor, and storage memory, as well as a means of communicating with another Internet-enabled device or the Internet directly. In addition, software including algorithms is necessary for the filtering, interpretation, organization, and storage of the collected raw data. Finally, a mobile phone or computer application is often employed to present the data to the user in real time or as a retrospective report. Some wearable technology providers allows the third party development of mobile or computer applications through the use of software development kits (SDK) or application programing interfaces (API).

## Conceptual Model of Wearable Technology Uses in Surgery

**Figure 1:**
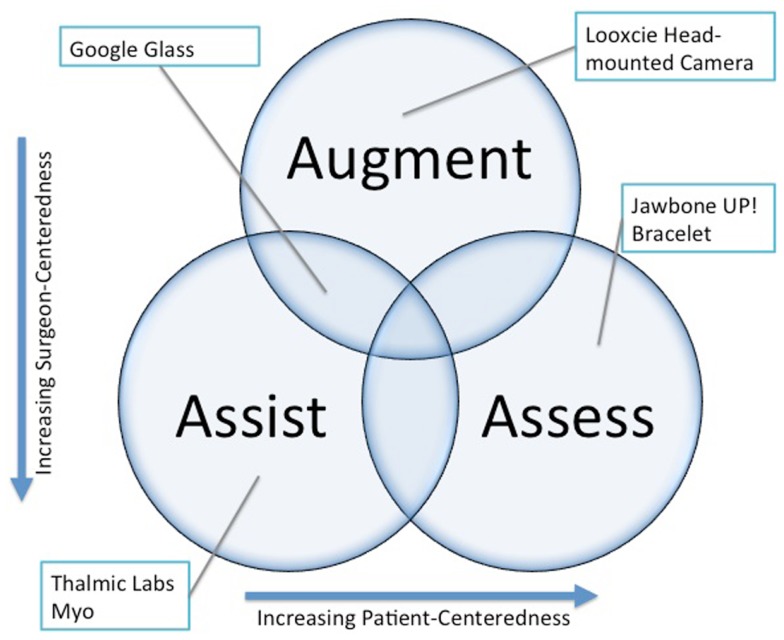
**Wearable technology functions within surgical practice**.

Conceptually, wearable technology can be deployed in a surgical practice in three main roles: assistance, augmentation, and assessment (Figure 1). A discussion of each general use case and specific examples is provided below. Considerable overlap exists between the roles, and one device can serve several simultaneously.

### Assistance

Assistance refers to the utilization of wearable technologies to replace physical tasks encountered in surgical practice. This can include tasks performed while in a sterile environment or tasks in clinic. An example of surgical task replacement would be the use of an arm-mounted device to allow gesture control of a PACS system to allow synchronized review of cross-sectional imaging at the time of surgery without breaking sterility (8). Another example from the clinic would be the capture of objective range-of-motion data utilizing sensors integrated into a hand-held device that the surgeon carries.

### Augmentation

Augmentation refers to the real-time provision of information to the surgeon during clinical or surgical encounters. The forms of information might include device or instrument data, clinical or biometric data, static reference material such as journal articles, or live communication with colleagues or others. In practice, this might take the form of a heads-up display of vital signs during cardiac surgery or a program, which allows recognition of specific instruments by operating room staff unfamiliar with complex equipment sets such as those used in orthopedic trauma surgery through visual recognition software. Superimposing diagnostic imaging such as a CT scan onto the operative field would fully realize the potential of image guided surgery. Context specific patient information might also be displayed during clinic encounters to improve the efficiency of workflow.

### Assessment

Assessment refers to the use of wearable technology in the objective measurement of disease severity or clinical outcomes and can also be extended to utilization of these technologies in surgical education. An example of outcomes measurement would be the tracking of breathing and sleep in patients before and after surgery to correct a deviated septum. An example of the definition of disease severity would be the quantification of mobility in patients with spinal stenosis over time in order to detect acute worsening of walking tolerance related to disease progression. This type of analysis has already been proposed in chronic obstructive lung disease monitoring (9) and stroke recovery (10). In surgical education, it would be possible to track the physiologic response of learners and educators to better understand stressors in real and simulated surgical environments.

## Routes of Wearable Technology Deployment

Surgeons can expect wearable technology to influence their practice in several broad ways: layered onto current practice, parallel innovations, and disruptive innovations (Table 1).

**Table 1:** **Modes of wearable technology innovation impacting the practice of surgery**.

Layered technology	Parallel technology	Disruptive technology
• Google glass as a PACS display	• Jawbone up activity data demonstrating decreased mobility in vasculopath	• Prediction of disease progression before symptoms based on biometric data
• Lumo control of table height in surgery based on posture	• Beddit employed to monitor improvements in sleep after rotator cuff surgery	• Continuous lactate measurement and reporting through transcutaneous skin patch

### Layered technologies

Layering implies the wearable technology is designed to fit within the current workflow of a practicing surgeon. Typically the development and deployment of such technologies stems from a practitioner repurposing an available device within their practice or collaboration between a developer and a practitioner. Currently, layered technologies dominate media reports of wearable technology application in surgery (include ref). This dominance relates to the surgeon-centric development of use cases, which consequently allows the surgeon to control any impact on their clinical practice. Specific examples include the development of gesture control for use in sterile environments or head-mounted cameras for live demonstrations of surgical procedures from the surgeon perspective.

### Parallel technologies

Parallel technologies encompass consumer-focused wearable technologies employed by patients that measure constructs, which are related to surgical practice. The data collected could be produced by a patient in a clinical encounter to substantiate a functional deficit or to demonstrate post-operative improvement. Activity trackers provide an example of a technology, which measures the mobility and sleep of patients, both of which are outcome metrics of many disciplines within surgery. The adoption of such data in current practice is heavily influenced by the practitioner as control of the data rests with the patient. A concrete example might be a lung transplant patient presenting data indicating declining physical activity to a thoracic surgeon.

### Disruptive technologies

The term disruptive innovation was originally coined by Clayton Christensen in 1995 and referred to technologies that replace an existing value network and create a new market (11). The invention of the Model-T Ford, a mass-produced, less expensive car replacing horse-drawn transportation is often given to describe this concept. A potential example of a disruptive application of wearable technology in surgical practice would be the use of a sensor-laden brace in rehabilitation after orthopedic surgery. Such a device could eliminate the need for post-operative visits to advance activity by providing constant communication of progress to surgeon and therapist. A more disruptive concept would be a device that could predict and prevent diseases. Such an example could take the form of a device able to sense the biometric signal leading to exacerbations of inflammatory bowel disease. Such a device could then allow users to link the biometric signature with associated activities in order to avoid them and prevent recurrences.

## Regulatory Barriers to Wearable Technology Integration in Surgical Practice

**Figure 2:**
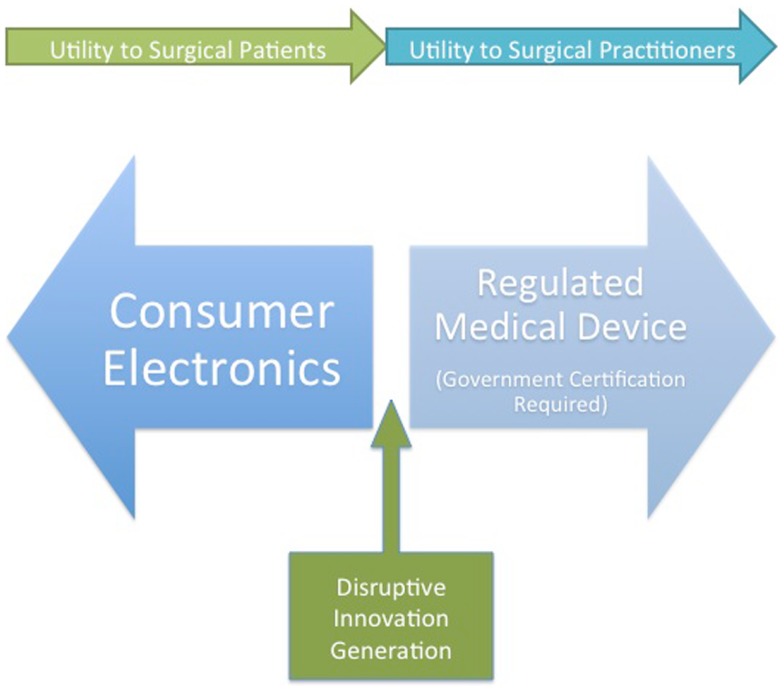
**A conception of wearable device developer strategies and the potential benefits for surgeons and surgical patients**. Disruptive innovation will likely occur at the junction between medical device and consumer electronics.

At the present moment, the majority of wearable technology is being developed for the consumer market. One of the main factors for the dearth of approved wearable devices in the medical field is the perceived hurdle of regulatory body approval. In addition, privacy and data ownership concerns serve as barriers to rapid development of surgically targeted devices. Many innovators in the space are taking advantage of the blurred line between device and consumer product to develop “near-medical” devices. This is the likely approach that will allow the development of disruptive technologies as opposed to the less nimble medical device world and will provide surgical patients with the most perceived benefit (Figure 2).

### Medical device and mobile medical application regulation

The Food and Drug Administration (FDA) regulates the sale of medical devices through the 510k process (12). To be defined as a medical device, a technology must be intended for diagnostic or therapeutic use.

Mobile medical applications (MMA) are also regulated under the FDA. Guidance on the definition of a regulated MMA was presented in a 2013 document released by the FDA (13). The guideline allows the development and use of applications by clinicians within their practice for medical applications, thereby giving physicians license to develop MMA, but not to sell or widely distribute prior to regulatory review as a medical device.

### Privacy and data ownership concerns

Regulations exist regarding the collection, maintenance, and transfer of protected health information by healthcare professionals and institutions. Independent parties collecting such information are not currently regulated by the health insurance portability and accountability act (HIPAA). However, if working with a medical institution or practitioner, third parties can necessitate the creation of a data use agreement. Little guidance exists on the necessity for a data use agreement when simply accepting collected data from a patient or using a device with video or sound capture in clinical situations.

## Limited Reimbursement Potential

Given the focus on the consumer market few comparisons of emerging wearable technology and incumbent medical devices exists. Until such a body of literature exists, insurers will not reimburse for wearable technologies used in clinical practice. Examples exist of pilot programs employing activity trackers to measure the physical activity of subscribers in order to provide rebates (14). This suggests the potential that insurers will provide reimbursement for prescribed technology in the future. The FDA medical device approval process has been shown to provide an avenue to successful reimbursement for the use of wearable technology (15).

## Surgeon Participation – The Path to Positive Impact on Surgical Patients

**Figure 3:**
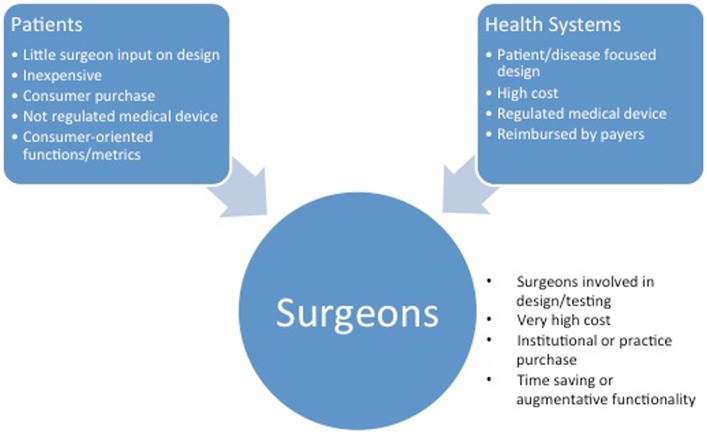
**The characteristics of innovations based on the intended customer**. Consumer- and hospital system-focused wearable technologies may appear in surgical practice by a push mechanism.

Given the bias toward the development of consumer use cases for the majority of current wearables, it is essential that practitioners assume a leadership role in the validation of medical applications (Figure 3). At present, surgeons primarily participate in the design and testing of devices aimed at increasing efficiency in practice or the operating room. Two main approaches are available to practitioners interested in applying wearable technology to their practice in a meaningful way: *de novo* development and repurposing.

*De novo* development implies working directly with hardware developers to create a device or application that addresses a specific clinical situation or need. A potential example would be the development of socks able to discern changes in force distribution in the foot of a diabetic patient due to poor footwear or a Charcot process.

Repurposing involves the utilization of an existing technology platform for the collection or presentation of data without custom configuration for the surgical environment. An example would be the use of a head-mounted camera for the filming of educational videos to be employed in surgical education.

## Potential Future Augmentation of Surgical Practice – Reimagining the Patient–Surgeon Relationship

Although it is easy to visualize heads-up display and gesture control augmenting the surgeon experience in the operating room, the objective assessment and presentation of user-generated data may provide a more transformative influence on surgical practice. In an era of patient-centered outcomes and shared decision-making the collection, interpretation, and communication of the biometrics allow patient and surgeon to better understand the impact of disease on the individual and provide the substrate for a discussion of treatment alternatives and expectations.

In the current paradigm of episodic care, the surgeon is like an air traffic controller who can only see airplanes when they are on the ground. As soon as they take off (i.e., leave the office or hospital) they are invisible, as are their symptoms and disabilities. Wearable technology, properly applied, can provide a more objective glimpse into the true nature of a patient’s illness allowing improvements in shared decision-making (16).

## Conclusion

Wearable technology can assist, augment, and provide a means of patient assessment for the surgeon. Current and future devices layer on existing practice patterns, operate in parallel or disrupt the current care paradigm. Regulatory and privacy concerns must be clarified to decrease the barriers to the utilization of wearable technology in surgery. Although assistive and augmentative technology will dominate the early application of wearables in surgery, these devices may eventually allow better communication and decision-making by surgeons through the collection, interpretation, and presentation of context relevant patient-specific measurements of surgical disease.

## Conflict of Interest Statement

Jesse Alan Slade Shantz is a paid employee of OMsignal, Inc., a biometric apparel company that has developed a line of shirts able to continuously track heart rate, heart rate variability, respirations, and movements and has stock or stock options of OMsignal, Inc.
